# Astrocyte-derived exosomal miR-378a-5p mitigates cerebral ischemic neuroinflammation by modulating NLRP3-mediated pyroptosis

**DOI:** 10.3389/fimmu.2024.1454116

**Published:** 2024-08-08

**Authors:** Ruiting Sun, Wenxin Liao, Ting Lang, Keyi Qin, Keyan Jiao, Le Shao, Changqing Deng, Yan She

**Affiliations:** ^1^ Medical School, Hunan University of Chinese Medicine, Changsha, China; ^2^ The First Hospital of Hunan University of Chinese Medicine, Changsha, China; ^3^ School of Integrated Chinese and Western Medicine, Hunan University of Chinese Medicine, Changsha, China

**Keywords:** astrocyte-derived exosomes, pyroptosis, inflammation, cerebral ischemia, NLRP3, miR-378a-5p

## Abstract

**Objective:**

This study aimed to investigate the regulatory role of astrocyte-derived exosomes and their microRNAs (miRNAs) in modulating neuronal pyroptosis during cerebral ischemia.

**Methods:**

Astrocyte-derived exosomes were studied for treating cerebral ischemia in both *in vitro* and *in vivo* models. The effects of astrocyte-derived exosomes on neuroinflammation were investigated by analyzing exosome uptake, nerve damage, and pyroptosis protein expression. High throughput sequencing was used to identify astrocyte-derived exosomal miRNAs linked to pyroptosis, followed by validation via qRT‒PCR. The relationship between these miRNAs and NLRP3 was studied using a dual luciferase reporter assay. This study used miR-378a-5p overexpression and knockdown to manipulate OGD injury in nerve cells. The impact of astrocyte-derived exosomal miR-378a-5p on the regulation of cerebral ischemic neuroinflammation was assessed through analysis of nerve injury and pyroptosis protein expression.

**Results:**

Our findings demonstrated that astrocyte-derived exosomes were internalized by neurons both *in vitro* and *in vivo*. Additionally, Astrocyte-derived exosomes displayed a neuroprotective effect against OGD-induced neuronal injury and brain injury in the ischemic cortical region of middle cerebral artery occlusion (MCAO) rats while also reducing pyroptosis. Further investigations revealed the involvement of astrocyte-derived exosomal miR-378a-5p in regulating pyroptosis by inhibiting NLRP3. The overexpression of miR-378a-5p mitigated neuronal damage, whereas the knockdown of miR-378a-5p increased NLRP3 expression and exacerbated pyroptosis, thus reversing this neuroprotective effect.

**Conclusion:**

Astrocyte-derived exosomal miR-378a-5p has a neuroprotective effect on cerebral ischemia by suppressing neuroinflammation associated with NLRP3-mediated pyroptosis.Further research is required to comprehensively elucidate the signaling pathways by which astrocyte-derived exosomal miR-378a-5p modulates neuronal pyroptosis.

## 1 Introduction

Ischemic stroke arises from severe insufficient blood supply to the brain, resulting in inadequate oxygen delivery and exacerbating neuronal cell death (1). The pathophysiology of cerebral ischemic nerve injury is multifaceted, with the inflammatory cascade playing a pivotal role throughout the progression of the condition. Consequently, the regulation of neuroinflammation has been identified as a crucial strategy for protecting against cerebral ischemic injury (2, 3). One form of programmed cell death closely associated with the neuroinflammation is pyroptosis. It can be initiated through various pathways, with the traditional pyroptosis pathway typically triggered by the NLRP3 inflammasome that activate cysteinyl aspartate specific proteinase 1 (Caspase 1) to cleave gasdermin D (GSDMD), resulting in pore formation and the subsequent release of proinflammatory mediators. Numerous studies have provided evidence that the regulation of pyroptosis plays a crucial role in neuroinflammation during ischemic stroke (4–6).

Astrocytes, which are widely distributed and contain numerous neuroglial cells in the central nervous system, play vital roles in sustaining neuroinflammation, participating in the immune response, controlling local blood flow, aiding in establishing the blood‒brain barrier, and regulating synaptic growth and plasticity (7, 8). Astrocytes exhibit robust secretory capabilities, and various pathological insults, including ischemia, hypoxia, inflammation, trauma, and tumors, induce astrocytes to secrete exosomes (9, 10). Astrocyte-derived exosomes serve as mediators of interactions with neurons and play a role in the regulation of neuroinflammation, ultimately contributing to the progression of cerebral ischemia (11–13). Exosomes, extracellular vesicles with diameters ranging from 30 to 150 nm, facilitate cell-to-cell communication by transporting bioactive substances such as DNA, RNA, and proteins (14–16). Current studies have shown the involvement of exosomes in the regulation of neural function through their miRNA cargo (17–19). However, further investigation is required to elucidate the role of astrocyte-derived exosomes in the modulation of neuroinflammation following cerebral ischemia.

In this study, primary astrocytes and neurons were isolated and cultured, and astrocyte-derived exosomes were extracted and identified. Our findings demonstrated that in both the rat MCAO model and the primary neuronal OGD model, astrocyte-derived exosomes were internalized by neurons and suppressed pyroptosis, ultimately leading to neuroprotective effects. Moreover, sequencing of isolated astrocyte-derived exosomes revealed that miR-378a-5p plays a significant role in the regulation of neuroinflammation. Subsequent dual luciferase reporter analysis demonstrated the presence of binding sites for miR-378a-5p on NLRP3-3 and URT. Astrocyte-derived exosomes were engineered to contain both a miR-378a-5p mimic and inhibitor, and subsequent research findings demonstrated that a miR-378a-5p inhibitor led to the induction of pyroptosis and exacerbated neuronal injury. This study enhances comprehension of the pathological mechanisms underlying cerebral ischemic neuroinflammation and offers valuable insights into the potential therapeutic strategies involving exosomes for treating neuroinflammation.

## 2 Methods

### 2.1 Isolation and cultivation of primary astrocytes and neurons

Astrocytes were isolated from the cortices of three-day-old Sprague‒Dawley (SD) rats. The quality certificate number ZS-202402200010 was provided by Hunan SJA Laboratory Animal Co., Ltd. Cells were cultured in DMEM/F12 basic medium (PM150312, Procell) supplemented with 10% fetal bovine serum (164210-50, Procell) and 1% penicillin‒streptomycin mixed solution (AWH0529a, Abiowell).

Cortical neurons were harvested from SD rats within 24 h of birth. The quality certificate number ZS-202401170023 was provided by Hunan SJA Laboratory Ani-mal Co., Ltd. Cultures were incubated in neurobasal medium (10888-022, Gibco) supplemented with 10% fetal bovine serum, 1% penicillin‒streptomycin mixed solution, 2% B27 supplement (17504-044, Gibco), and 0.25% L-glutamine supplement (35050-061, Gibco) for 4 to 8 h before they were transferred to fetal bovine serum-free neuronal medium.

### 2.2 Extraction and identification of astrocyte-derived exosomes

Astrocyte-derived exosomes were isolated through ultracentrifugation. Astrocyte supernatants were collected and centrifuged at 300 ×g for 15 min to remove any dead cells, after which the cell fragments were removed via centrifugation at 12,000 ×g for 30 min. The resulting supernatant was ultrafiltered and concentrated via centrifugation at 3,000 ×g for 30 min, after which the exosomes were precipitated via ultracentrifugation at 120,000 ×g for 90 min. Subsequently, after being resuspended in PBS, the exosomes were centrifuged at 120,000 ×g for an additional 90 min for purification.

Transmission electron microscopy (TEM) was utilized to analyze the morphology of astrocyte-derived exosomes, while nanoparticle tracking analysis (NTA) was employed to assess the particle size range of the exosomes. Additionally, the presence of the surface marker proteins CD81 and TSG101 on exosomes was detected through Western blotting.

### 2.3 Establishment of the OGD model

The original neuron culture medium was replaced with DMEM supplemented with sugar-free basic culture medium (PM150270, Procell), and the cells were subjected to sugar deprivation and hypoxia by incubating them in a three-gas incubator at 37°C, 5% CO2, 1% O2, and 94% N2.

### 2.4 Establishment of the MCAO model

Healthy SPF SD rats, with an equal distribution of males and females weighing between 260 g and 280 g, were selected. The quality certificate number ZS-202401230013 was obtained from Hunan SJA Laboratory Animal Co., Ltd. The rats in the experimental group were given a 1% pentobarbital sodium intraperitoneal injection to induce unconsciousness, and the right common carotid artery was exposed through a median cervical incision. The proximal end of the right carotid artery was clamped, followed by an incision at its bifurcation to insert an embolus toward the right internal carotid artery, thereby inducing occlusion of the middle cerebral artery on one side. After confirming the completion of ischemia, the outer embolus was retracted until resistance was felt, indicating that it had reached the incision site of the common carotid artery; thus, MCAO modeling was completed. In the sham operation group, the rats underwent blood vessel separation without any vascular ligation or introduction of a suture embolus. The animal experimentation process adhered to the principles and requirements outlined in the Guidelines for Ethical Review of Laboratory Animal Welfare (GB/T 35892:2018).

### 2.5 Labeling and uptake of exosomes

The PKH-26 linker (UR52302, Umibio) or the PKH-67 linker (UR52303, Umibio) was combined with Diluent C at a 1:9 ratio to create the working solution. The purified exosomes were labeled by adding the working solution and then incubating at room temperature for 10 min, followed by centrifugation at 120000 ×g for 90 min to eliminate excess dye. The neurons were exposed to astrocyte-derived exosomes (30 µg/mL), and exosome uptake was visualized using fluorescence microscopy after a total incubation period of 24 h.

### 2.6 Cell viability

Neuronal viability was evaluated using a CCK8 (C0038, Beyotime) assay. Neurons were cultured in 96-well plates at 3 × 104 cells per well, and 10 µL of CCK8 solution was added to every 100 µL of neuron culture medium. Using a microplate reader, the absorbance at 450 nm (A450) was determined after 2 h of incubation at 37°C. The following formula was used to determine cell viability: cell viability = (OD experimental group - OD blank group)/(OD control group - OD blank group).

### 2.7 Detection of lactate dehydrogenase activity

The release rate of lactate dehydrogenase (LDH) was used to detect neuronal injury. LDH release in the neuronal supernatant was examined using an LDH cytotoxicity detection kit (C0017, Beyotime), and the absorbance at 490 nm (A490) was measured utilizing an enzyme labeling instrument. The LDH release level in the control group was established as 100%, and values in the other groups were normalized to this benchmark.

### 2.8 Immunofluorescence

Cells or tissues were exposed to primary antibodies against GFAP (1:100, 16825-1-AP, Proteintech), NSE (1:800, PA1-10010, Invitrogen), GSDMD (1:100, 20770-1-AP, Proteintech), or NLRP3 (1:100, PAB38738, Bioswamp) at 4°C overnight. The sections were then treated with fluorescent secondary antibodies at room temperature for 1 h following three PBS washes. After three rinses with PBS, DAPI staining solution was applied, and the plate was sealed with an anti-fluorescence attenuation plate sealing agent before fluorescence microscopy scanning imaging. Fluorescence positive cell expression rate (%) = number of fluorescent labeled cells/total number of cells(DAPI) ×100%.

### 2.9 Nissl staining

Following 6 h of cerebral ischemia in SD rats, the brain tissue was fixed and sectioned. These sections were dehydrated, immersed in xylene and ethanol solutions, and then soaked in distilled water for dewaxing. Nissl dye was used for staining, and subsequent rinsing with distilled water removed any excess coloration. A differentiation solution (1% glacial acetic acid) was then applied to distinguish the cellular components, and microscopic observation was initiated once the background became colorless with distinct cell contours. Finally, buffer glycerin was used to seal the sections before further microscopic examination. ImageJ image analysis software was used to calculate the gray value of Nissl bodies within neurons located in the cortical region of the rat brain.

### 2.10 Cell transfection

The miR-378a-5p mimics and miR-378a-5p inhibitors were acquired from Su-zhou GenePharma Co., Ltd. Neurons containing astrocyte-derived exosomes (30 µg/mL) were transfected with the green fluorescent protein-labeled lentiviral vector LV3 (H1/GFP&Puro) at an MOI = 100 for 48 h before being subjected to OGD treatment for further experimentation.

### 2.11 Western blot analysis

The total protein content of astrocyte-derived exosomes, neurons, or the cerebral cortex was extracted using RIPA lysis buffer supplemented with PMSF and quantified utilizing a BCA protein concentration assay kit. After separation by 12% gel electrophoresis, the membrane was transferred to a polyvinylidene fluoride (PVDF) membrane and blocked with 5% skim milk powder for 2 h. The membrane was then incubated overnight at 4°C with primary antibodies against CD81 (1:1000, ab109201, Abcam), TSG101 (1:1000, ab125011, Abcam), GAPDH (1:10,000, 81649-5-RR, Proteintech), GSDMD (1:1000, AF4012, Affinity), NLRP3 (1:1000, PAB38738, Bioswamp), Caspase-1 p20 (1:1000, AF4005, Affinity), and β-actin (1:2000, 20536-1-AP, Proteintech). Subsequently, the membrane was washed with TBST and incubated for 2 h at room temperature with the secondary antibody. Subsequently, the ECL luminescent liquid gel imaging display strips were subjected to thorough cleaning.

### 2.12 QRT‒PCR

TRIzol was used to extract total RNA from the cells or tissues, and the total RNA was quantified at OD 260/OD280. Following the manufacturer’s instructions, cDNA was reverse transcribed using the NovoScript^®^ miRNA First-Strand cDNA Synthesis Kit and SYBR qPCR Kit (E172-YH01, Novoprotein). The sequences of primers used are provided below, and data analysis was conducted using the 2^–△△Ct^ method (**Supplementary Table 1**).

### 2.13 Analysis of the dual-luciferase reporter gene

Dual luciferase reporter gene analysis was employed to confirm the binding sites between miR-378a-5p and the NLRP3 3’-UTR. 293T cells were cultured in 12-well plates and transfected with either wild-type psiCHECK-2-NLRP3-WT or mutant psiCHECK-2-NLRP3-MUT rat NLRP3 gene double luciferase reporter plasmids (obtained from HonorGene) in conjunction with miR-378a-5p negative control (NC) or miR-378a-5p mimic (procured from Sangon Biotech) using a Lip2000 Transfection Reagent (Invitrogen, USA). The luminescence signals of firefly and Renilla luciferases were quantified using a chemiluminescence detector.

### 2.14 Statistical analysis

GraphPad Prism (version 9.0) was utilized for the statistical analysis, and all the data are presented as the means ± standard deviations. One-way analysis of variance, was used to compare groups according to a statistically significant threshold of *P*<0.05, while *P*<0.01 indicated statistical significance.

## 3 Results

### 3.1 Identification of primary astrocytes, neurons, and astrocyte-derived exosomes

**Figure 1:**
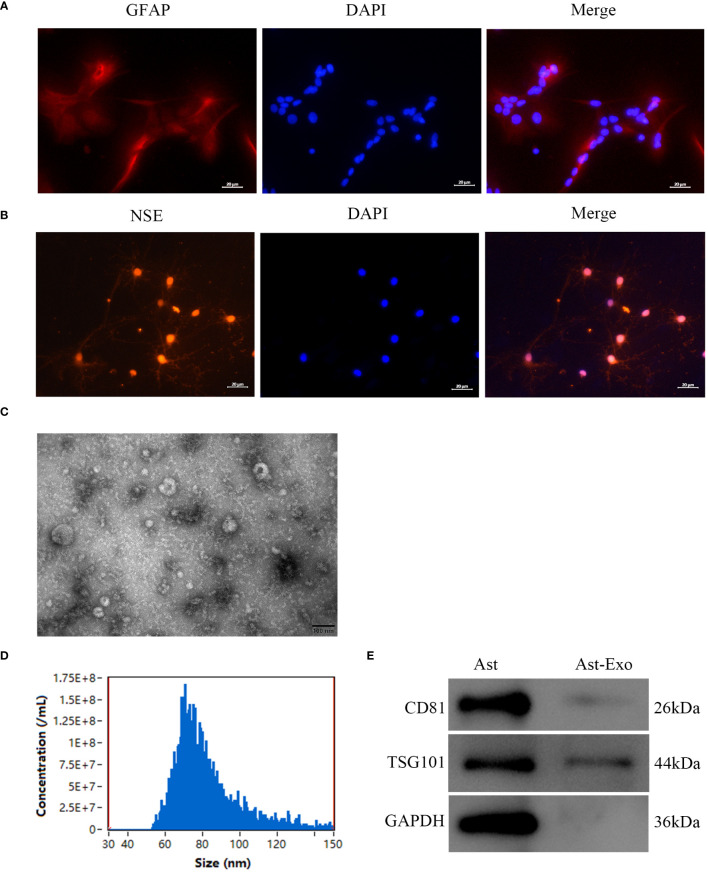
Identification of primary astrocytes, neurons, and astrocyte-derived exosomes was performed as follows. **(A)** Immunofluorescence assay of astrocytes revealed the presence of the marker GFAP. Scale bar = 20 µm. **(B)** Immunofluorescence staining was employed to detect the presence of the neuron marker NSE. Scale bar = 20 µm. **(C)** TEM was used to visualize exosomes produced from astrocytes. Scale bar = 100 nm. **(D)** The particle size distribution of astro-cyte-derived exosomes was evaluated using NTA. **(E)** Exosome marker protein expression levels were determined by Western blot analysis utilizing CD81 and TSG101.

Within the central nervous system, glial fibrillary acidic protein (GFAP) acts as a distinctive marker for differentiating astrocytes from other glial cells. Immunofluorescence staining revealed prevalent cytoplasmic GFAP expression in star-shaped, fusiform, and polygonal astrocytes, which feature large oval nuclei and thick, elongated synapses (**Figure 1A**). Neuron-specific enolase (NSE), a marker of neurons, is expressed in both the cytoplasm and cell membrane of neurons. Additionally, the growth of axons and dendrites was prominently evident, as they formed an intricate network structure (**Figure 1B**). TEM revealed that the astrocyte-derived exosomes exhibited a characteristic “cup-and-dish” morphology, with a bilayer structure (**Figure 1C**). NTA revealed that the distribution of astrocyte-derived exosomes ranged from 30 to 150 nm in diameter (**Figure 1D**). Western blot analysis confirmed the presence of the surface marker proteins CD81 and TSD101 on the astrocyte-derived exosomes (**Figure 1E**).

### 3.2 Astrocyte-derived exosomes are internalized by neurons subjected to OGD and mitigate OGD-induced neuronal damage

**Figure 2:**
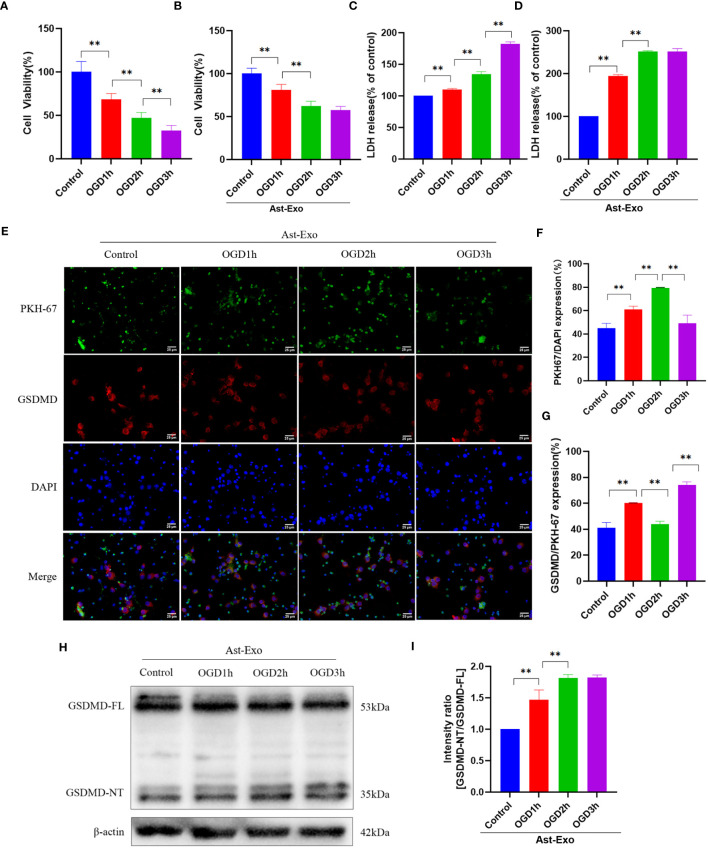
Astrocyte-derived exosomes were internalized by OGD neurons and conferred protection against OGD-induced neuronal injury. **(A, B)** Cell viability of neurons was assessed using CCK8 in the presence or absence of astrocyte-derived exosomes. **(C, D)** LDH release rate was measured in neurons with or without astrocyte-derived exosomes. **(E-G)** Immunofluorescence staining was used to assess the uptake of exosomes from astrocytes and the manifestation of neuronal pyroptosis protein GSDMD. Scale bar = 25 µm. **(H, I)** GSDMD expression of the neuronal pyroptosis protein was measured using the Western-Blot method. The statistical data for every group is presented as the mean ± SD (n≥ 3). *P < 0.05, ** *P* < 0.01.

The results from the CCK-8 and LDH release experiments indicated a notable reduction in neuronal cell viability and an increase in cell injury with prolonged OGD (**Figures 2A, C**). However, the introduction of astrocyte-derived exosomes during the period from OGD 2h to OGD 3h did not further aggravate neuronal injury (**Figures 2B, D**). Exosome uptake was detected using PKH-67, which emits green fluorescence. Coexpression of GSDMD, indicated by red fluorescence, suggested the occurrence of pyroptosis in neurons upon exosome uptake. The results demonstrated a significant increase in the amount of astrocyte-derived exosomes taken up by neurons after OGD 2h (*P*<0.01), followed by no decrease in the level of the pyroptosis protein GSDMD (*P*<0.01) (**Figures 2E–G**). Further examination revealed no discernible difference in GSDMD pyroptosis protein expression between the OGD 2h group and the OGD 3h group (**Figures 2H, I**). In conclusion, our speculation is that astrocyte-derived exosomes exert neuroprotective effects by mitigating further neuronal damage during OGD 2h.

### 3.3 Astrocyte-derived exosomes mitigate OGD-induced neuronal pyroptosis

**Figure 3:**
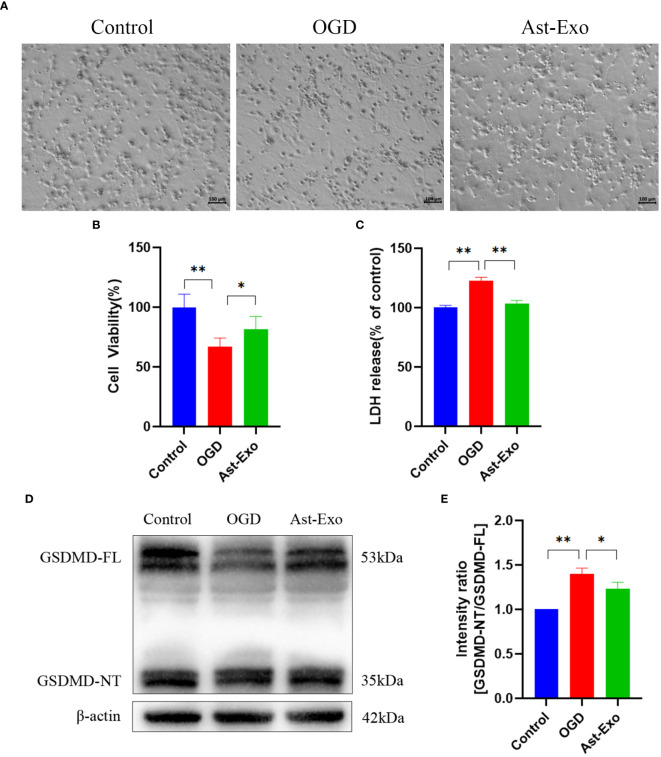
Astrocyte-derived exosomes attenuate OGD-induced neuronal pyroptosis. **(A)** Morphological observation of neurons. Scale bar = 100 µm. **(B)** The cell viability of neurons was assessed using CCK8. **(C)** LDH release rate of neurons. **(D, E)** GSDMD expression of the neuronal pyroptosis protein was measured using the Western-Blot method. The statistical data for every group is presented as the mean ± SD (n ≥ 3). *P < 0.05, ** *P* < 0.01.

We further elucidated the impact of astrocyte-derived exosomes on neuronal pyroptosis induced by OGD 2 h through morphological analysis, CCK-8 assays, LDH measurements, and GSDMD protein evaluation. Upon microscopic examination, the control group displayed robust neuronal cell bodies with densely interwoven axons and dendrites, forming a network. In contrast, the OGD group presented with faint neuronal cell bodies, along with fragmented axons and dendrites, accompanied by a decrease in neuron count. Nevertheless, the administration of astrocyte-derived exosomes alleviated damage to axons and dendrites while preventing a decrease in the neuron population (**Figure 3A**). The results from the CCK-8 and LDH assays indicated a significant improvement in neuronal viability (*P*<0.05) and a noteworthy reduction in cell injury (*P*<0.01) following exosome treatment (**Figures 3B, C**). We further found that the expression of pyroptosis protein GSDMD increased after OGD (*P*<0.01), which was subsequently mitigated by astrocyte-derived exosomes (*P*<0.05) (**Figures 3D, E**).

### 3.4 Astrocyte-derived exosomes enhance neuroprotection and attenuate pyroptosis in a rat model of MCAO

**Figure 4:**
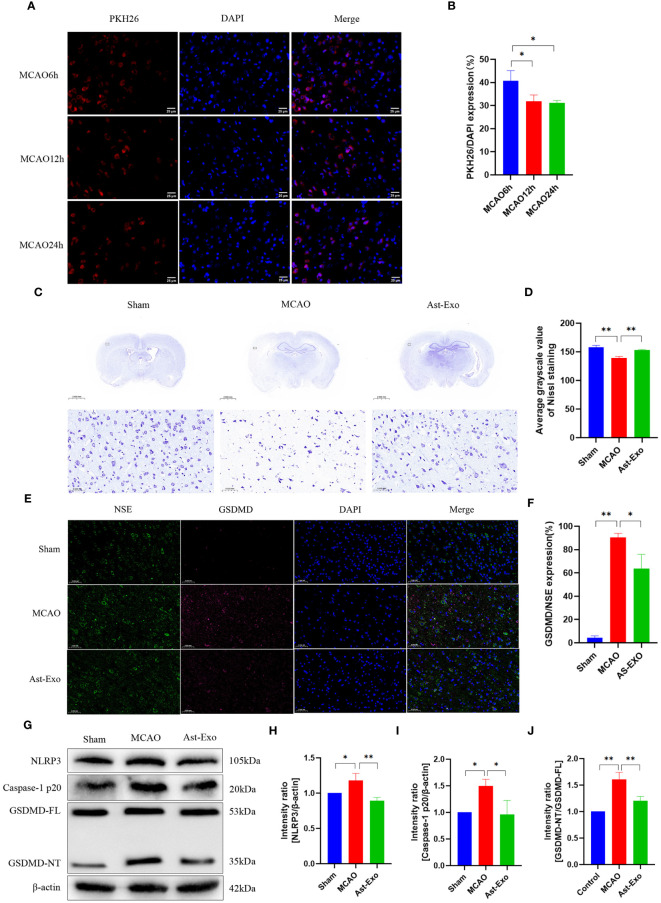
Astrocyte-derived exosomes enhanced brain injury recovery and attenuated pyroptosis in rats subjected to MCAO. **(A, B)** Fluorescence imaging revealed the entry of PKH26-labeled astrocyte-derived exosomes into the brain. Scale bar = 25 µm. **(C, D)** Nissl staining was performed to assess neuronal survival. **(E, F)** Immunofluorescence staining demonstrated the expression of GSDMD, a key protein involved in pyroptosis, in cortical neurons on the ischemic side of rats. Scale bar = 50 µm. **(G-J)** The amounts of GSDMD, Caspase-1 p20, and NRP3 proteins in the cortical region on the ischemic side were assessed by Western blot analysis. The statistical data for every group is presented as the mean ± SD (n ≥ 3). *P < 0.05, ** *P* < 0.01.

Moreover, animal experiments were conducted to further validate the role of astrocyte-derived exosomes in pyroptosis of cerebral ischemic. PKH-26-labeled astrocyte-derived exosomes were observed in the rat brain at 6 h, 12 h, and 24 h after tail vein injection. The findings revealed that the cellular uptake of exosomes was most notable at the 6-h time point, hence, a subsequent experimental time of 6 h after exosome injection was selected (**Figures 4A, B**). Nissl staining revealed that neurons in the cerebral cortex of the sham group displayed structural integrity, uniform staining, and a tightly organized arrangement. Conversely, following cerebral ischemia, shallow staining was observed in the cerebral cortex, accompanied by a significant reduction in cell count, a sparse and disordered arrangement, the appearance of vacuoles, and a notable decrease in Nissl bodies. However, the administration of astrocyte-derived exosomes substantially increased both the number of neurons and the number of Nissl bodies (**Figures 4C, D**). NSE serves as a reliable biomarker for neuronal cells. The coexpression of red fluorescent GSDMD and green fluorescent NSE indicated neuronal pyroptosis. The results revealed a notable increase in GSDMD and NSE coexpression in the MCAO group (*P*<0.01), while astrocyte-derived exosome intervention led to a decrease in their coexpression (*P*<0.05) (**Figures 4E, F**). Western blot analysis revealed a notable increase in the protein expression of NLRP3, cleaved caspase-1, and GSDMD in the MCAO group (*P*<0.05). Conversely, following astrocyte-derived exosome intervention, there was a notable decrease in the protein expression of NLRP3, cleaved caspase-1, and GSDMD (*P*<0.01) (**Figures 4G–J**). In conclusion, astrocyte-derived exosomes exert neuroprotective effects by alleviating MCAO-induced brain injury and reducing pyroptosis.

### 3.5 Astrocyte-derived exosomal miR-378a-5p contributes to neuroprotection through targeted regulation of NLRP3

**Figure 5:**
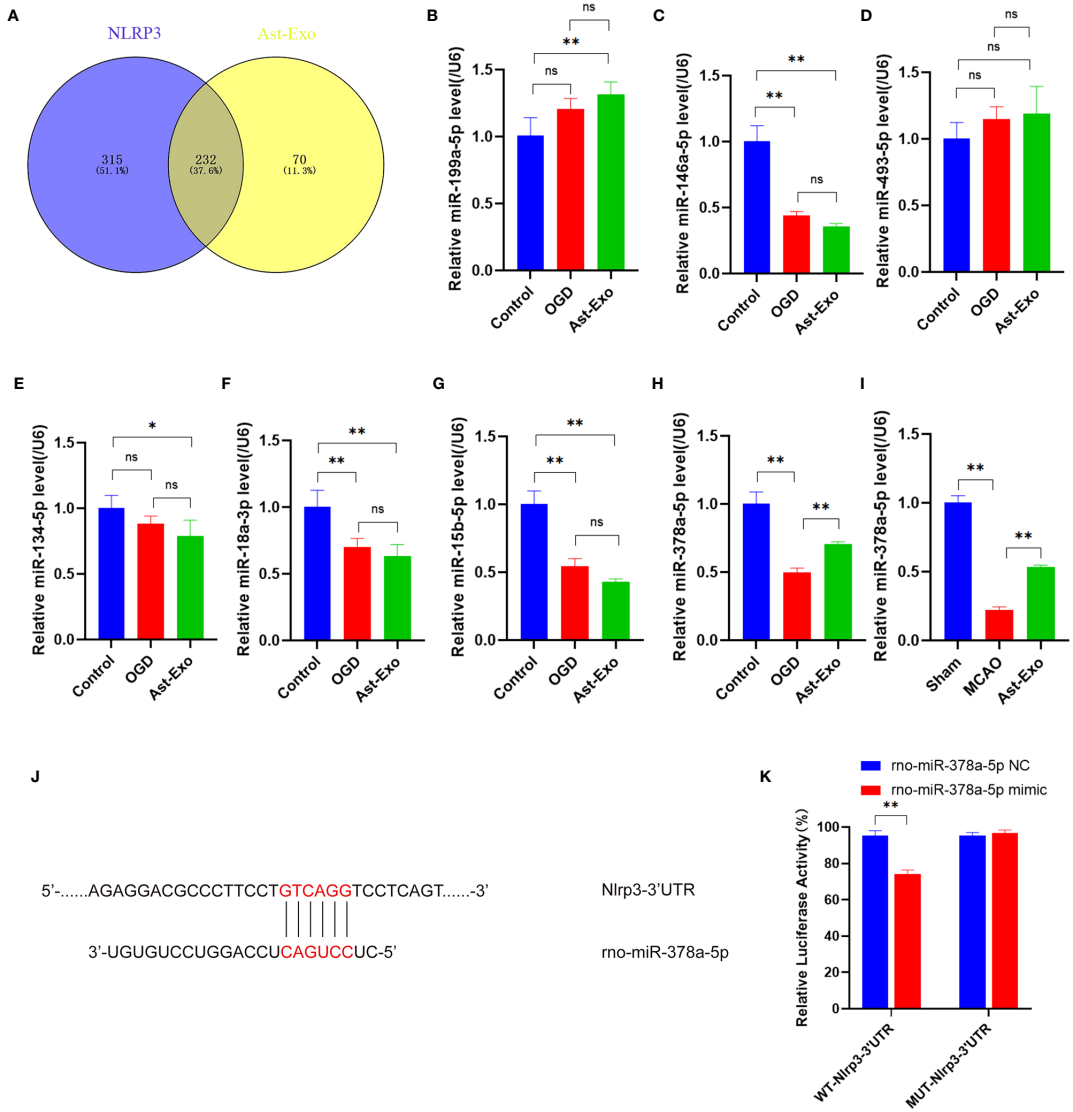
Astrocyte-derived exosomal miR-378a-5p contributes to neuroprotection by targeting NLRP3. **(A)** The miRNAs from astrocyte-derived exosomes were compared with NLRP3-related miRNAs in the miRWalk database. **(B-I)** The miRNA expression was assessed using qRT-PCR. **(J, K)** Dual-luciferase assay was performed to confirm the specific binding site between miR-378a-5p and NLRP3 3'UTR. The statistical data for every group is presented as the mean ± SD (n≥ 3). *P < 0.05, ** *P* < 0.01.

We sequenced astrocyte-derived exosomes and identified 232 miRNAs associated with NLRP3 (**Figure 5A**). Moreover, among these 232 miRNAs, 7 were closely linked to pyroptosis, namely, miR-199a-5p, miR-146a-5p, miR-493-5p, miR-134-5p, miR-18a-3p, miR-15b-5p and miR-378a-5p. The quantitative real-time PCR (qRT‒PCR) results revealed obvious upregulation of only miR-378a-5p following treatment with astrocyte-derived exosomes, while no significant differences were detected for the other miRNAs. Subsequent *in vivo* verification demonstrated that astrocyte-derived exosomes upregulated miR-378a-5p expression within the brain cortex on the ischemic side of the rats (**Figures 5B–I**). The public database TargetScan was utilized for the prediction of potential binding sites between miR-378a-5p and NLRP3, while a dual-luciferase reporter assay confirmed the presence of a highly unique and conserved sequence between the NLRP3 3’UTR and miR-378a-5p (**Figure 5J**). Our results demonstrated that in cells transfected with the WT-Nlrp3 3’-UTR reporter plasmid, transfection with miR-378a-5p mimics significantly decreased luciferase activity. However, transfection with the MUT-Nlrp3 3’UTR reporter plasmid had no impact on luciferase activity (**Figure 5K**).

### 3.6 Astrocyte-derived exosomal miR-378a-5p mitigates neuronal pyroptosis following cerebral ischemia by suppressing the expression of NLRP3

**Figure 6:**
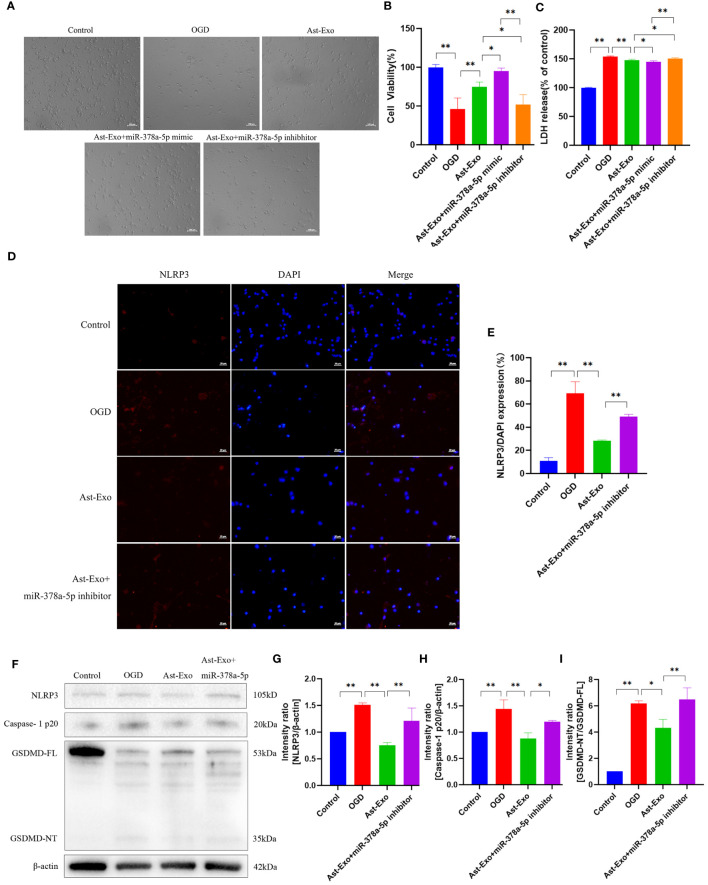
Astrocyte-derived exosomal miR-378-5p attenuates neuronal pyroptosis after cerebral ischemia by down-regulating NLRP3. **(A)** Morphological observation of neurons. Scale bar = 100 µm. **(B)** CCK8 assay for neuronal cell viability. **(C)** LDH release rate of neurons. **(D, E)** NLRP3 expression of neurons detected by immunofluorescence. Scale bar = 20 µm. **(F-I)** Western-Blot assay for neuronal pyroptosis proteins NLRP3, Caspase-1p20 and GSDMD. The statistical data for every group is presented as the mean ± SD (n > 3). *P < 0.05, ** *P* < 0.01.

We further examined the effect of miR-378a-5p overexpression and knockdown in astrocyte-derived exosomes on OGD-induced neurons. Our results demonstrated that the upregulation of miR-378a-5p effectively attenuated neuronal synaptic damage and cell death, notably increasing cell viability and decreasing cellular injury. Conversely, reduced levels of miR-378a-5p exacerbated neural damage (**Figures 6A–C**). NLRP3 was visualized using red fluorescence, and immunofluorescence analysis revealed that astrocyte-derived exosomes attenuated the expression of NLRP3 under OGD conditions (*P*<0.01). In contrast, the knockdown of miR-378a-5p in astrocyte-derived exosomes promoted the expression of NLRP3 (*P*<0.01) (**Figures 6D, E**). Western blot analysis revealed that astrocyte-derived exosomes notably attenuated the expression of NLRP3, cleaved caspase-1, and GSDMD under OGD conditions (*P*<0.01). In contrast, silencing miR-378a-5p in astrocyte-derived exosomes notably elevated the expression levels of NLRP3, cleaved caspase-1, and GSDMD (*P*<0.01) (**Figures 6F–I**). Overall, these results underscore the neuroprotective role of astrocyte-derived exosomes delivering miR-378a-5p, which alleviates OGD-induced neuronal injury and pyroptosis by suppressing the NLRP3 signaling pathway and ameliorating cerebral ischemia.

## 4 Discussion

The central mechanism underlying neuroinflammation involves the activation of glial cells, astrocytes, which constitute the majority of glial cells in the brain. After a cerebral ischemic event, astrocytes exhibit dual functions in regulating neuroinflammation. Overactive astrocytes induce the activation and recruitment of numerous inflammatory cells through the release of various proinflammatory cytokines or chemokines, initiating an inflammatory cascade response following cerebral ischemia (20, 21). In contrast, astrocytes have the capacity to release a diverse array of reparative neurotrophic factors, enhance neuronal viability, safeguard the integrity of the blood‒brain barrier, suppress inflammatory responses, and ameliorate neurological impairment following cerebral ischemia (22, 23). Astrocytes serve as key modulators of neuroinflammation and are essential for preserving internal homeostasis following cerebral ischemia (24, 25). Nonetheless, the mechanisms through which astrocytes interact with neurons to modulate neuroinflammation remain inadequately understood.

Exosomes are extracellular vesicles with a diameter of 30-150 nm. The formation process involves the initial inward folding of the plasma membrane into intracellular vesicles, which then develop into multivesicular bodies. Upon fusion with the plasma membrane, these bodies are released into the extracellular space, where they can fuse with other plasma membranes to perform their functions (26, 27). Astrocyte-derived exosomes facilitate the transmission of information from donor cells to peripheral nerve cells, enabling intercellular communication and material exchange within the central nervous system. This process plays a crucial role in targeted transportation, regulation of target cell function, and overall intercellular information exchange in the central nervous system. Consequently, astrocyte-derived exosomes serve as pivotal “communication messengers” in central nervous system diseases (28, 29).The intervention of astrocyte-derived exosomes in the presence of the inflammatory factor IL-1β enhances neuronal uptake of astrocyte-derived exosomes, suggesting that inflammatory conditions may facilitate intercellular communication between astrocyte-derived exosomes and neurons (30). Astrocyte-derived exosomes can induce neuroprotective effects through the modulation of inflammatory signaling pathways (10, 17, 18, 31, 32). Nevertheless, additional validation is required to ascertain the potential association between the regulation of neuroinflammation by astrocyte-derived exosomes and pyroptosis. In this study, our observations indicate that astrocyte-derived exosomes exert neuroprotective effects by preventing neuronal damage during prolonged OGD. Subsequent animal studies demonstrated that astrocyte-derived exosomes enhanced neurological function and MCAO rats while also diminishing pyroptosis. Thus, our findings from both *in vivo* animal models and *in vitro* cell models provide evidence supporting the neuroprotective effects of astrocyte-derived exosomes on cerebral ischemia.

MiRNAs serve as pivotal mediators of intercellular communication in astrocyte-derived exosomes, with these exosomes acting as both carriers and reservoirs of abundant miRNAs. miRNAs, which are composed of 21 to 23 nucleotides, are endogenous noncoding RNAs that are widely distributed among higher organisms and exhibit high evolutionary conservation. Upon entering the cytoplasm of target cells, exosomal miRNAs specifically associate with the 3’-untranslated region of corresponding mRNAs, thereby modulating the expression of target genes and exerting crucial biological functions (30–33). By virtue of their miRNA cargo, astrocyte-derived exosomes play a vital role in signal transduction and regulation during the pathological process of inflammatory injury following cerebral ischemia (34–36). In this study, based on our previous observation that astrocyte-derived exosomes can mitigate OGD-induced neuronal pyroptosis, we sequenced astrocyte-derived exosomes and identified miRNAs associated with pyroptosis. MiRNAs in astrocyte-derived exosomes were sequenced, and 232 miRNAs associated with NLRP3 were identified. Among these, 7 miRNAs were implicated in the regulation of pyroptosis. Subsequent PCR validation confirmed the association of miR-378a-5p with the regulation of neuronal injury. NLRP3 plays a pivotal role in the modulation of pyroptosis (37–39). We conducted dual luciferase assays to investigate the interaction between miR-378a-5p and NLRP3, revealing the presence of binding sites between the two molecules. Subsequently, Astrocyte-derived exosomes were engineered to contain a miR-378a-5p mimic and inhibitor, and their impact on neuronal activity was investigated. Overexpression of miR-378a-5p in astrocyte-derived exosomes was found to enhance neuronal activity, while knockdown of miR-378a-5p exacerbated neuronal damage and increased the expression of NLRP3-related pyroptosis proteins. These findings suggest that astrocyte exosome-delivered miR-378a-5p may have a neuroprotective effect on cerebral ischemia by suppressing NLRP3-mediated pyroptosis and reducing neuroinflammation.

A small non coding RNA that regulates gene expression horizontally after transcription by miR-378, It has two mature chains: miR-378a-3p and miR-378a-5p. Concurrently, Clinical studies have revealed that targeting miR-378a-5p holds significant importance for the auxiliary diagnosis and treatment of diverse diseases. The research indicates that miR-378a-5p can serve as a potential companion diagnostic for abiraterone acetate in patients with metastatic castration-resistant prostate cancer, thereby monitoring the efficacy of the drug over time (40). MiR-378a-5p participates in the epigenetic regulation of SUFU gene expression in placental tissue through targeting, thereby influencing intrauterine growth restriction caused by placental insufficiency (41). Additionally, LINC00514 can enhance the proliferation and invasion of esophageal squamous cell carcinoma cells by sponging miR-378a-5p, and facilitate the progression of esophageal squamous cell carcinoma (42). MiR-378a-5p inhibits GZMB expression in children with acetaminophen overdose-induced liver injury (43). MiR-378a-5p has been shown to decrease the proliferation of rectal cancer cells through the inhibition of its target gene CDK1 (44). Furthermore, Bone marrow mesenchymal stem cells-derived exosomes containing miR-378a-5p have been demonstrated to alleviate symptoms of rheumatoid arthritis (45). However, at present, there are limited studies on the role of miR-378a-5p in cerebral ischemia, miR-378a-5p has been found to reduce neuronal apoptosis by downregulating the expression of CAMKK2, thereby mitigating cerebral ischemia‒reperfusion injury. MiR-378a-5p has been found to reduce neuronal apoptosis by downregulating the expression of CAMKK2, thereby mitigating cerebral ischemia‒reperfusion injury (46). Our research has discovered, for the first time, that astrocyte-derived exosomal miR-378a-5p regulates the neuroinflammatory pathological process of cerebral ischemic pyroptosis.

This study furnishes a novel theoretical foundation for exploring the treatment of cerebral ischemic neuroinflammatory injury and offers an experimental research basis for the drug development of exosome miRNAs as targets in clinical practice. Nevertheless, the study presented certain limitations. The mechanism pathway through which miR-378a-5p inhibits NLRP3 has not been investigated yet, thus it is indispensable to further explore it in the future.

## 5 Conclusion

**Figure 7:**
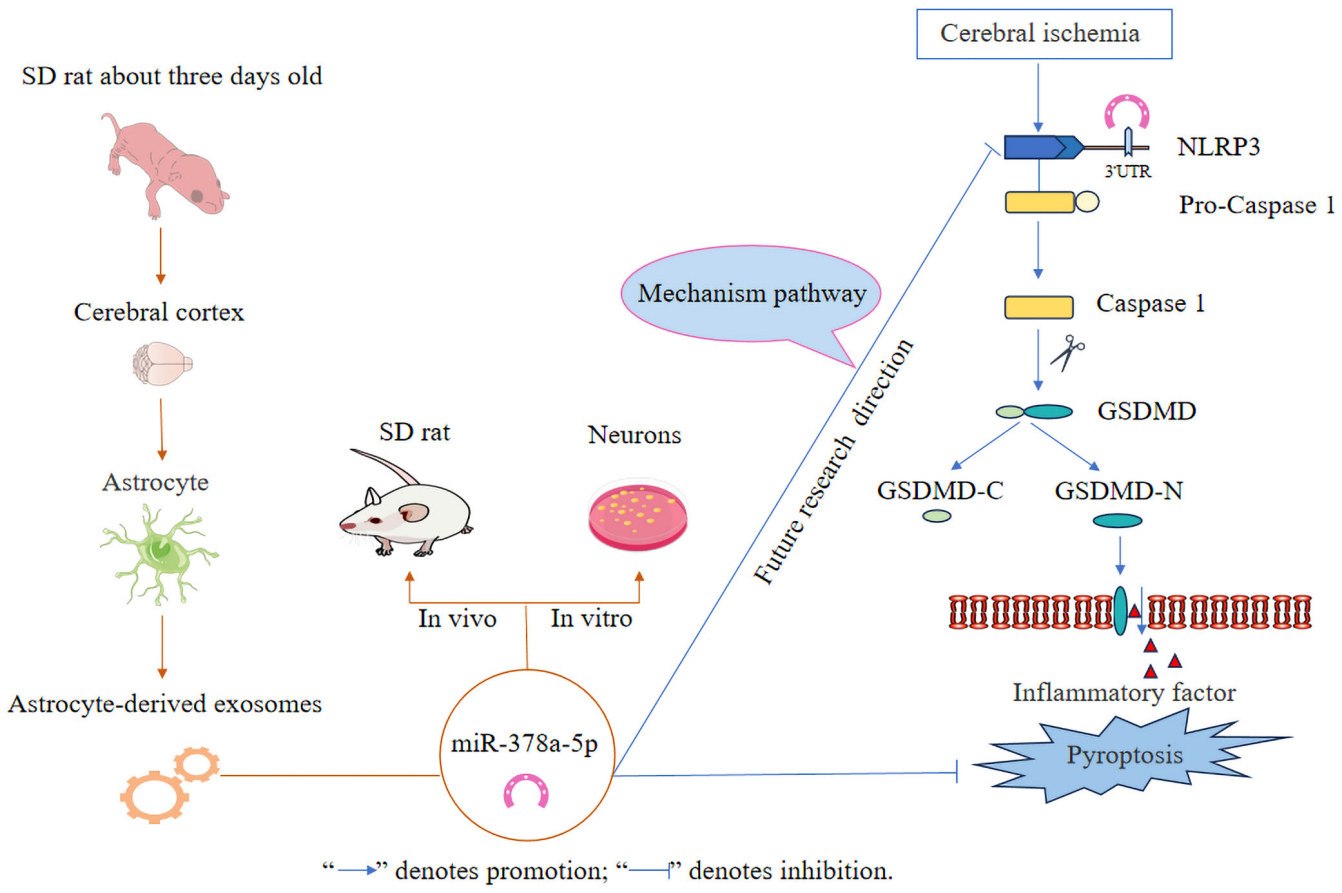
Schematic illustration of astrocyte-derived exosomal miR-378a-5p mitigates cerebral ischemic neuroinflammation by modulating NLRP3-mediated pyroptosis.

The innovative findings of this study are as follows: (1) Astrocyte-derived exosomes have neuroprotective effects by preventing nerve cell injury and alleviating neuronal pyroptosis induced by OGD and MCAO injury. (2) Astrocyte-derived exosomal miR-378a-5p mitigates cerebral ischemic neuroinflammation through the inhibition of NLRP3-mediated pyroptosis. Future research will concentrate on elucidating the mechanistic pathways of astrocyte-derived exosomal miR-378a-5p in cerebral ischemic neuroinflammation, as depicted in **Figure 7**.

## Data availability statement

The original contributions presented in the study are included in the article/**Supplementary Material**. Further inquiries can be directed to the corresponding authors.

## Ethics statement

The animal study was approved by Animal Ethics Committee at Hunan University of Chinese Medicine. The study was conducted in accordance with the local legislation and institutional requirements.

## Author contributions

RS: Methodology, Software, Validation, Writing – original draft, Writing – review & editing. WL: Software, Validation, Writing – original draft. TL: Validation, Writing – original draft. KQ: Visualization, Writing – review & editing. KJ: Software, Writing – original draft. LS: Writing – review & editing, Software. CD: Conceptualization, Methodology, Writing – review & editing. YS: Conceptualization, Methodology, Writing – review & editing.
