# Supplementary material for: Astrocyte-derived exosomal miR-378a-5p mitigates cerebral ischemic neuroinflammation by modulating NLRP3-mediated pyroptosis
Source: Front Immunol. 2024 Aug 8;15:1454116. doi: 10.3389/fimmu.2024.1454116 (PMC11338813; doi:10.3389/fimmu.2024.1454116)
Supplement: Supplementary file 1 [file Table_1.docx]

Supplementary Material

Astrocyte-derived exosomal miR-378a-5p mitigates cerebral ischemic neuroinflammation by modulating NLRP3-mediated pyroptosis

Ruiting Sun1†, Wenxin Liao1†, Ting Lang1, Keyi Qin1, Keyan Jiao1, Le Shao2, Changqing Deng1*, Yan She1*

*** Correspondence:** Changqing Deng: dchangq@hnucm.edu.cn; Yan She: 003199@hnucm.edu.cn

## Supplementary Tables

| **Gene** | **Forward primer sequence (5’-3’)** |
| --- | --- |
| miR-378a-5p | GGCGCTCCTGACTCCAGGTC |
| miR-199a-5p | CGCGCCCAGTGTTCAGACTAC |
| miR-146a-5p | AGCGCGTGAGAACTGAATTCCA |
| miR-493-5p | CAGCGCGTTGTACATGGTAGGCT |
| miR-134-5p | CGCGTGTGACTGGTTGACCA |
| miR-18a-3p | CGCGACTGCCCTAAGTGCT |
| miR-15b-5p | AGCGCGTAGCAGCACATCATG |
| U6 | CTCGCTTCGGCAGCACA |

**Supplementary Tables 1.** Sequence of the forward primer for qRT-PCR
